# The Protective Effects of N-Acetylserotonin Against Cisplatin-Induced Renal Injury: A Biochemical and Histopathological Study

**DOI:** 10.3390/ijms27093896

**Published:** 2026-04-27

**Authors:** Selçuk Yazıcı, Gülay Turan, Merve Akış Yılmaz, Büşra Aslan Akyol, Caner Yıldırım, Oğuzhan Korkut

**Affiliations:** 1 Department of Pediatrics, Faculty of Medicine, Bezmialem University, 34093 Istanbul, Turkey; 2 Department of Pathology, Faculty of Medicine, Balıkesir University, 10145 Balıkesir, Turkey; gulay.tr@yahoo.com.tr; 3 Department of Biochemistry, Faculty of Medicine, Balıkesir University, 10145 Balıkesir, Turkey; merve.akis@balikesir.edu.tr; 4 Department of Veterinary Pharmachology and Toxicology, Institute of Health Sciences, Balıkesir University, 10145 Balıkesir, Turkey; busraslan26@gmail.com; 5 Department of Biochemistry, Institute of Health Sciences, Balıkesir University, 10145 Balıkesir, Turkey; dytcaneryildirim@gmail.com; 6 Department of Clinical Pharmachology, Faculty of Medicine, Balıkesir University, 10145 Balıkesir, Turkey; ogkorkut@hotmail.com

**Keywords:** N-acetyl-5-hydroxytryptamine, cisplatin-induced renal injury, malondialdehyde, superoxide dismutase, total oxidant status, total antioxidant status

## Abstract

Cisplatin is a potent chemotherapeutic agent whose clinical application is frequently limited by severe nephrotoxicity. N-acetylserotonin (NAS), a precursor of melatonin and a selective agonist of the TrkB receptor, has demonstrated significant antioxidant and neuroprotective properties. This study aimed to evaluate the potential renoprotective effects of NAS against cisplatin-induced acute kidney injury (AKI) in a rat model. Thirty-five Wistar Albino rats were divided into five groups: Control, Sham, NAS (5 mg/kg), Cisplatin (CP; 7.5 mg/kg), and CP + NAS. NAS was administered daily for seven days, while cisplatin was given as a single dose on the fourth day. Renal function was assessed via serum urea and creatinine. Oxidative stress markers, including Malondialdehyde (MDA), Superoxide Dismutase (SOD), Total Antioxidant Status (TAS), and Total Oxidant Status (TOS), were measured in kidney tissue. Comprehensive histopathological evaluations were performed to assess tubular and glomerular damage. Cisplatin administration significantly increased serum creatinine levels and induced severe histopathological damage (*p* < 0.05). While cisplatin reduced SOD and TAS levels, NAS treatment showed a trend toward biochemical recovery without reaching statistical significance in oxidative markers. Notably, NAS administration significantly ameliorated cisplatin-induced histopathological lesions, specifically reducing tubular epithelial loss, glomerular degeneration, interstitial inflammation, and vacuolization (*p* < 0.05). Our findings indicate that NAS exerts a profound structural protective effect against cisplatin-induced renal injury. The preservation of renal parenchyma, despite modest systemic biochemical shifts, suggests that NAS-mediated protection may involve localized TrkB-dependent pro-survival signaling and stabilization of mitochondrial integrity. NAS represents a promising therapeutic candidate for mitigating chemotherapy-induced nephrotoxicity.

## 1. Introduction

Reactive Oxygen Species (ROS) are highly reactive, oxygen-containing molecules, including free radicals such as the superoxide anion (O_2_^−^) and non-radicals like hydrogen peroxide (H_2_O_2_), primarily produced as byproducts of aerobic metabolism within the mitochondrial electron transport chain [1,2]. While essential for processes such as redox signaling and innate immunity, their overproduction triggers oxidative stress. This stress leads to cellular damage by attacking and modifying vital biomolecules through initiating lipid peroxidation, which degrades cellular membranes; oxidatively modifying proteins to alter their function; and damaging DNA, potentially leading to mutations [2]. Enzymatic antioxidants, including superoxide dismutase (SOD), catalase (CAT), and glutathione peroxidase (GPx), alongside non-enzymatic antioxidants such as thiols and reduced glutathione (GSH), play pivotal roles in protecting cells against ROS-induced damage [3,4].

The chemotherapy drug cisplatin is a major cause of nephrotoxicity, often presenting as dose-limiting acute kidney injury (AKI). The primary histopathological manifestations are acute tubular necrosis (ATN) and apoptosis within the kidney’s proximal tubules, where the drug preferentially accumulates [5,6]. Cisplatin-induced toxicity involves complex mechanisms, including direct cytotoxicity, oxidative stress, mitochondrial dysfunction, inflammation, and apoptosis [5]. In addition, cisplatin has been shown to induce multi-organ toxicity affecting the liver, nervous system, and gastrointestinal tissues, primarily mediated by oxidative damage and inflammatory pathways [7].

Malondialdehyde (MDA) is a key secondary end-product of lipid peroxidation and a widely used biomarker for assessing oxidative damage. An elevation in MDA concentration is interpreted as a direct indicator of pathological lipid peroxidation and oxidative stress in tissues [8]. Superoxide Dismutase (SOD) is a crucial antioxidant enzyme that converts the superoxide radical into the less reactive hydrogen peroxide, serving as a primary enzymatic defense line [9]. Total Antioxidant Status (TAS) and Total Oxidant Status (TOS) are comprehensive biochemical parameters used to evaluate the overall balance between oxidants and antioxidants. TOS provides an overall measure of all ROS, indicating the cumulative level of oxidation in tissue. Conversely, TAS reflects the combined action of both enzymatic and non-enzymatic antioxidant defense systems. Concomitant changes, such as a decrease in TAS and an increase in TOS, effectively indicate severe tissue damage [10].

N-Acetylserotonin (NAS) (chemical name: N-Acetyl-5-hydroxytryptamine), or normelatonin, is a natural intermediate in the biosynthesis of melatonin from serotonin. Beyond its role as a precursor, NAS acts as an agonist for melatonin and tropomyosin receptor kinase B (TrkB) receptors. The activation of TrkB is linked to its potential antidepressant effects, promotion of neurogenesis, and neuroprotective properties in tissues such as the retina and hippocampus. Furthermore, NAS is a potent antioxidant, with some studies suggesting it may be more effective than melatonin in scavenging free radicals and protecting against oxidative damage, particularly lipid peroxidation [11,12,13]. Although NAS is both the precursor and a metabolite of melatonin, it exerts several effects independent of melatonin against oxidative stress [12].

As the precursor molecule of melatonin, NAS has been associated with various pleiotropic effects. NAS exhibits antioxidant [12], anti-aging [14,15], neuroprotective [12], and antidepressant [15] properties, while also mitigating cognitive impairment [15] and lipid peroxidation [16,17]. Furthermore, antihypertensive and antitumor activities have been reported [18]. Protective effects have been documented in various tissues; specifically, NAS has been found to ameliorate damage in the brain [19,20], retina [21], liver [16,19,22], testes [23], bone [19], intervertebral disc [4], and kidney [14].

Melatonin and its metabolites exhibit antioxidative, anti-inflammatory, and anti-apoptotic effects. There are three identified melatonin receptors in mammals (MT1, MT2, and MT3). MT3 is found in the highest concentrations in the kidney and liver, and to a lesser extent in the heart, brain, and adipose tissue. MT3 is a cytosolic reductase enzyme possessing a melatonin-binding site; it has been identified as quinone reductase 2 (QR2) and inhibits the electron transfer reactions of quinones, thus playing a role in protecting the cell from oxidative stress. Notably, it has a higher binding affinity for NAS than for melatonin [19].

Considering that MT3 receptors are abundant in the kidney and exhibit a higher binding affinity for NAS than melatonin, this study aimed to investigate the effects of the potent antioxidant NAS on cisplatin-induced renal injury.

## 2. Results

### 2.1. Malondialdehyde (MDA)

**Figure 1:**
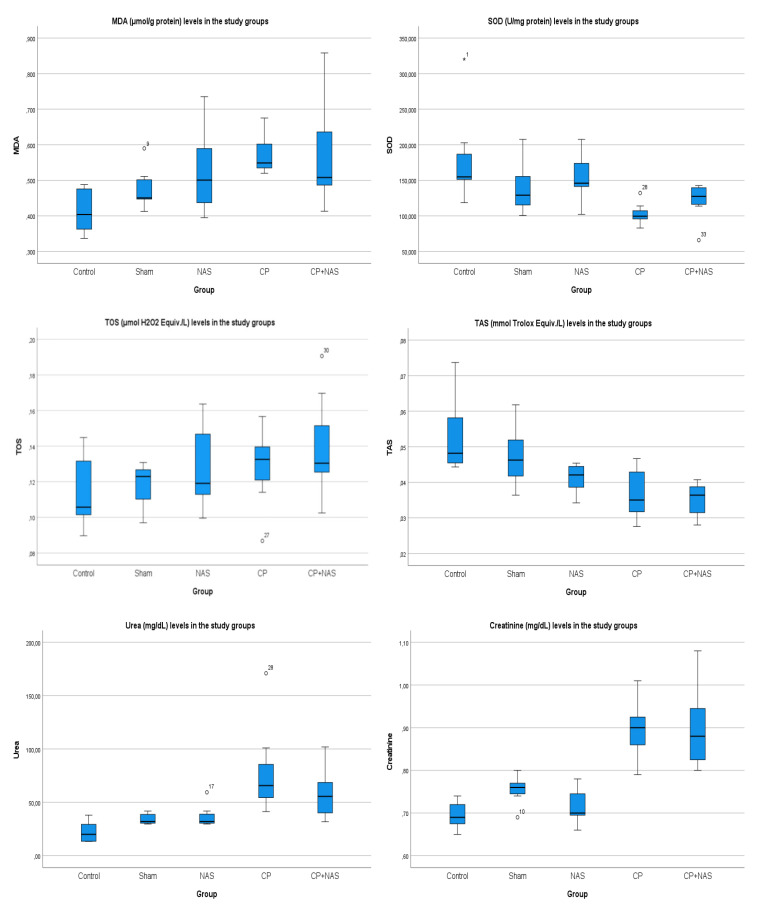
Boxplot graphs of MDA (μmol/g protein), SOD (U/mg protein), TOS (μmol H_2_O_2_ Equiv./L), TAS (mmol Trolox Equiv./L) urea (mg/dL), and creatinine (mg/dL) values in the study groups.

MDA levels in the kidney tissue were significantly higher in both the CP group (*p* < 0.001) and the CP + NAS group (*p* < 0.001) compared to the control group. No significant difference was observed between the sham, NAS, and control groups. Furthermore, MDA levels did not differ significantly between the CP and CP + NAS groups (Table 1, Figure 1).

### 2.2. Superoxide Dismutase (SOD)

SOD activities were not significantly different among the C, S, NAS, and CP + NAS groups. However, SOD levels were significantly lower in the CP group compared to the control group (*p* < 0.01). Although SOD levels were slightly higher in the CP + NAS group compared to the CP group, this difference did not reach statistical significance (Table 1, Figure 1).

### 2.3. Total Oxidant Status (TOS)

No significant difference was detected in TOS levels among the five experimental groups studied.

### 2.4. Total Antioxidant Status (TAS)

TAS levels did not differ significantly between the C, S, and NAS groups. Conversely, TAS was significantly lower in the CP (*p* < 0.01) and CP + NAS (*p* < 0.01) groups compared to the control group. TAS levels were also significantly lower in the CP group compared to the sham group (*p* < 0.05). Although TAS levels showed an upward trend in the CP + NAS group relative to the CP group, the difference was not statistically significant (Table 1, Figure 1).

### 2.5. Urea and Creatinine

Serum urea levels were highest in the CP group and lowest in the control group; however, these differences between the study groups were not statistically significant.

In contrast, serum creatinine levels did not differ between the C, S, and NAS groups. Creatinine concentrations in the CP group were significantly higher than those in the C (*p* = 0.001), S (*p* < 0.05), and NAS (*p* < 0.01) groups. Similarly, creatinine values in the CP + NAS group were significantly higher than in the C (*p* < 0.05), S (*p* < 0.05), and NAS (*p* < 0.05) groups. No significant difference in creatinine levels was observed between the CP and CP + NAS groups.

### 2.6. Pathological Examination Results

There was no significant difference between the control, sham, and NAS groups regarding any of the six histopathological parameters. The composite scores for all histopathological parameters in the CP group were significantly higher than those in the C, S, and NAS groups. Similarly, the total scores in the CP + NAS group were significantly higher than those in the C, S, and NAS groups.

Notably, scores for tubular epithelial loss (*p* < 0.05), glomerular degeneration (*p* < 0.05), interstitial inflammation (*p* = 0.01), and vacuolization (*p* < 0.05) were significantly higher in the CP group compared to the CP + NAS group, indicating a protective effect of NAS treatment. However, intraluminal cast formation and glomerular degeneration scores did not differ significantly between the CP and CP + NAS groups. Total histopathological scores are summarized in Table 2.

Overall, while NAS treatment resulted in significant histopathological improvement, biochemical parameters showed only limited and non-significant recovery, indicating a dissociation between structural and functional outcomes.

**Figure 2:**
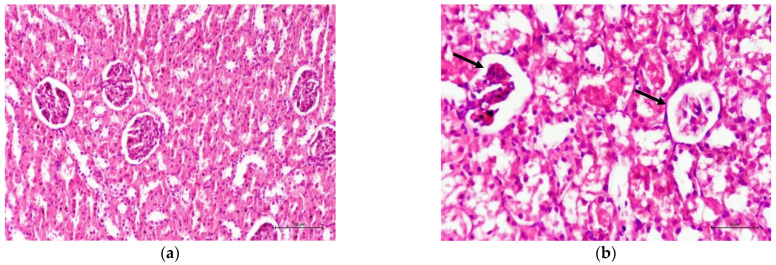

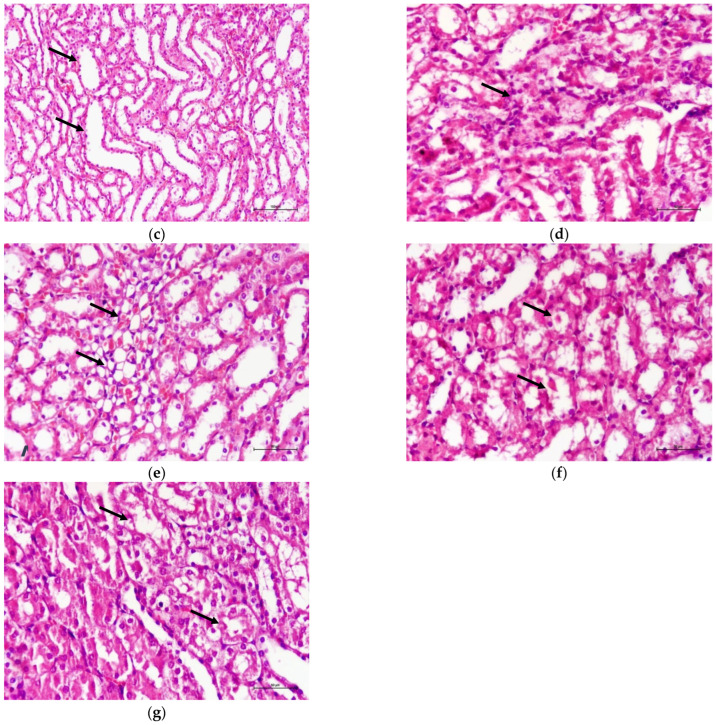
Images of histopathological findings obtained in pathological evaluations. (**a**) Glomeruli and tubules in the control group, hematoxylin and eosin, ×200. (**b**) Glomerular degeneration in the CP group, hematoxylin and eosin, ×400. (**c**) Tubular dilatation in the CP group, hematoxylin and eosin, ×200. (**d**) Interstitial inflammation in the CP group, hematoxylin and eosin, ×400. (**e**) Vacuolization in the CP group, hematoxylin and eosin, ×200. (**f**) Cast formation in the CP group, hematoxylin and eosin, ×400. (**g**) Tubular epithelium loss in the CP group, hematoxylin and eosin, ×40.

Representative images of histopathological findings from the renal tissue are presented in Figure 2.

## 3. Discussion

In the present study, NAS demonstrated significant histopathological protection against cisplatin-induced renal injury. However, this protective effect was not accompanied by equally robust biochemical recovery. This discrepancy suggests that structural preservation may precede measurable functional restoration.

The previous literature has highlighted the tissue-specific variability of NAS. Gesing et al. reported that NAS (20 mg/kg for 3 weeks) effectively attenuated MDA levels in the liver, brain, lungs, and testes in an aflatoxin B1-induced model; however, no effect was observed in the kidney as the toxin failed to induce lipid peroxidation in that specific tissue. Conversely, Oxenkrug et al. demonstrated that long-term, higher-dose oral NAS administration (20 mg/kg for 4 weeks) significantly reduced renal MDA levels, exhibiting potent antioxidant and anti-aging activity [14]. Similarly, Qingzhi Li et al. showed that NAS dose-dependently reduced hippocampal MDA elevation in a traumatic brain injury (TBI) model, where a 30 mg/kg dose successfully improved neurogenesis and mitigated cognitive impairments [24]. While NAS has also been shown to reduce iron-induced lipid peroxidation in testicular tissue [25], it did not significantly modulate MDA levels or demonstrate a lipid peroxidation-reducing effect at the specific dose and duration utilized in our study.

Interestingly, NAS slightly increased SOD levels in the CP + NAS group. The lack of difference in SOD levels between the CP + NAS and NAS-only groups is pivotal, as it suggests that NAS may directly enhance the antioxidant capacity within our renal injury model. Although not extensively studied in kidney tissues, NAS has shown corrective effects on MDA and SOD levels in liver cells under hydrogen peroxide-induced stress. This protection has been attributed to the upregulation of endogenous antioxidants and the suppression of mitochondria-dependent apoptotic pathways [2]. Yu et al. similarly found that 5 mg/kg NAS significantly ameliorated hepatic ischemia–reperfusion injury by modulating MDA and SOD levels [26]. While our study did not mirror these biochemical outcomes in terms of TOS or TAS differences, the robust structural preservation we observed suggests that NAS operates through pathways that prioritize cellular architecture over immediate systemic biochemical markers. Although NAS is widely recognized for its antioxidant properties [11,12], the absence of statistically significant changes in oxidative stress markers in this study indicates that antioxidant mechanisms may not be the primary pathway under these experimental conditions. Therefore, conclusions regarding antioxidant involvement should be interpreted cautiously.

The most salient finding of our study was the profound corrective effect of NAS on tubular epithelial loss, glomerular degeneration, interstitial inflammation, and vacuolization in CP-induced renal injury. These findings align with previous reports indicating that the predominant toxic effects of CP occur within the tubules, specifically the proximal segments [6,27]. The findings suggest that the renal protective effect of NAS may occur through multiple pathways. NAS inhibits mitochondrial death pathways and autophagic activation in the brain, but these effects have not been studied in kidney tissue [28]. While NAS studies have primarily focused on neuronal tissues, its nephroprotective potential is becoming increasingly apparent. The structural restoration observed here is particularly significant, as it indicates a localized preservation of the renal parenchyma.

It is well-established that the precursor (serotonin) and the subsequent metabolite (melatonin) of NAS possess protective effects in renal damage. Serotonin receptor activation stimulates mitochondrial biogenesis and homeostasis in renal injury [29], while melatonin exerts anti-inflammatory effects by reducing IL1-alpha, IL1-beta, Mcp-1 and TGF-beta1mRNA levels, thereby mitigating ROS formation, fibrosis, and apoptosis [30,31,32]. NAS appears to provide a unique bridge between these pathways.

Crucially, NAS is a potent and unique agonist of tropomyosin receptor kinase B (TrkB), the high-affinity receptor for Brain-Derived Neurotrophic Factor (BDNF) [1,33]. Unlike serotonin or melatonin, NAS rapidly activates TrkB, initiating downstream pro-survival cascades such as the PI3K/AKT and Ras-ERK pathways [33,34]. These pathways are essential for promoting cell survival and inhibiting apoptosis. In neuronal models, NAS has been shown to increase BCL-2 expression while decreasing BAX and cleaved caspases [4,13]. Furthermore, both melatonin and NAS have been found to inhibit mitochondrial death pathways, including the release of cytochrome c and Smac [19,28].

The significance of our findings is underscored by the widespread expression of TrkB receptors in the kidney, including proximal/distal tubule epithelial cells, the juxtaglomerular apparatus, and podocytes [35,36,37]. TrkB signaling in the kidney plays dual roles in maintaining renal function and ensuring proper structural development [37,38,39]. The pronounced histopathological improvement observed in our study—even in the absence of significant biochemical shifts—suggests that NAS targets these TrkB-dependent pro-survival properties to stabilize mitochondrial integrity and prevent cellular apoptosis in vulnerable renal tissues. The dose-dependent nature of NAS and its tissue-specific efficacy may explain the limited biochemical response in this study [4,16]. Factors such as tissue lipid content and the density of MT3/QR2 receptors likely influence the antioxidant potential of NAS in the renal environment [40]. The fact that NAS effectively halted the progression of visible tissue damage (epithelial loss and inflammation) positions it as a promising candidate for renoprotective therapy. The main limitation of our study is that the observed protective effects were not investigated across a range of doses to fully delineate the molecular mechanisms. Future research should focus on the dose–response relationship to determine if higher concentrations might also trigger the expected biochemical shifts alongside the observed structural benefits. However, these mechanisms were not directly investigated in the present study and should therefore be considered hypothetical.

This study has several limitations. First, only a single dose and short observation period were used. Second, mechanistic pathways were not directly investigated. Third, nutritional and inflammatory biomarkers (such as albumin and cytokines) were not assessed. Finally, comparisons with other renal injury models were not performed. These limitations should be considered when interpreting the findings. A further limitation is the reliance on histopathological findings despite the lack of concordant improvement in biochemical and functional markers, indicating a dissociation between structural preservation and measurable renal function, which warrants cautious interpretation of the observed renoprotective effects.

## 4. Materials and Methods

### 4.1. Experimental Procedure and Administered Drugs/Chemicals

This study was conducted at Balıkesir University’s Animal Experiments Laboratory, and the animals were obtained from the same facility. All experimental procedures were performed by researchers certified in animal experimentation.

A total of 35 two-month-old Wistar Albino rats (mean weight: 250 g, range: 190–280 g) were used. The rats were housed at room temperature (22–23 °C) under a 12 h light/dark cycle for an acclimatization period of 7 days, with 7 rats per cage. They had free access to food and water.

Rats were randomly assigned to five experimental groups (7 per group):

1. Control group (C): Received no treatment.
2. Sham group (S): Received an intraperitoneal (i.p.) injection of 0.5 mL of isotonic 1.2% ethanol solution daily (09:00 p.m.).
3. NAS group: Received daily i.p. injections of 5 mg/kg [2,4,19,26] N-acetylserotonin (NAS) dissolved in 0.5 mL of isotonic 1.2% ethanol solution.
4. Cisplatin group (CP): Received daily i.p. injections of 0.5 mL isotonic 1.2% ethanol solution, and a single dose of 7.5 mg/kg cisplatin [5,41,42] was administered i.p. on the fourth day.
5. CP + NAS group: Received daily i.p. injections of 5 mg/kg NAS dissolved in 0.5 mL of isotonic 1.2% ethanol solution, plus a single i.p. injection of 7.5 mg/kg cisplatin on the fourth day.

The selected NAS dose (5 mg/kg) was based on previous studies demonstrating tissue-protective effects at this dose while minimizing excessive drug exposure. The cisplatin dose (7.5 mg/kg) was chosen in accordance with established acute kidney injury models. All compounds were administered intraperitoneally to ensure consistent systemic exposure. Cisplatin was administered on day 4 to allow assessment of peak renal injury, which typically occurs within 72 h following administration [43,44].

### 4.2. Termination of the Experiment and Collection of Blood and Tissue Samples

Six hours after the final procedures on the seventh day, the rats were anesthetized with ketamine. Following laparotomy and thoracotomy, 5 mL of blood was collected from each animal via intracardiac aspiration. Both kidneys were rapidly dissected; the right kidneys were fixed for pathological examination, while the left kidneys were stored for biochemical analysis.

### 4.3. Supply of Kits Used

Kidney tissue homogenates were used to measure levels of total oxidant status (TOS; μmol H_2_O_2_ Equiv./L), total antioxidant status (TAS; mmol Trolox Equiv./L), superoxide dismutase (SOD; U/mg protein), and malondialdehyde (MDA; μmol/g protein). Serum samples were analyzed for urea (mg/dL) and creatinine (mg/dL).

N-acetyl-5-hydroxytryptamine (NAS, CAS No. 1210-83-9; also known as normelatonin) was obtained from Sigma-Aldrich (St. Louis, MO, USA). Cisplatin injectable solution (Cipintu^®^) was obtained from Onko Koçsel Pharmaceuticals (Kocaeli, Turkey). All other chemicals used for biochemical assays were of analytical grade and obtained from commercial sources.

### 4.4. Serum Sample Preparation

Serum samples were incubated at room temperature for 30–40 min and then centrifuged at 1500× *g* for 20 min at 4 °C. Aliquoted serum samples were stored at −80 °C until analysis.

### 4.5. Renal Function Test Analysis

To assess renal function, serum urea and creatinine levels were analyzed via colorimetric methods using a Mindray BS-400 autoanalyzer (Shenzhen Mindray Bio-Medical Electronics Co., Ltd., Shenzhen, China).

### 4.6. Preparation of Kidney Tissue Homogenates

Kidney tissues frozen at −80 °C were homogenized for MDA, SOD, TAS, and TOS analysis. First, tissues were washed with cold phosphate-buffered saline (PBS; 0.01 M, pH 7.4). Subsequently, tissues were weighed and cold PBS was added (0.9 mL per 0.1 g of tissue) to obtain a 10% (*w*/*v*) starting homogenate. Homogenization was performed using a Stuart SHM1 homogenizer (Cole-Parmer, Nottingham, UK). The homogenates were centrifuged at 10,000× *g* for 10 min at 4 °C (SIGMA 3-30K, Sigma Laborzentrifugen GmbH, Osterode am Harz, Germany). The resulting supernatants were separated and stored at −80 °C. Total protein concentration in the supernatants was determined using the Bradford reagent (Clearband, Eco-Tech, Ho Chi Minh City, Vietnam).

### 4.7. Biochemical Analyses

MDA levels were measured using a metabolic analysis kit (Elabscience, Cat. No. E-BC-K025-M, Houston, TX, USA) based on the thiobarbituric acid (TBA) method. The absorbance was measured at 532 nm using a Varioskan Flash Multimode Reader (Thermo Scientific, Waltham, MA, USA). Results are expressed as μmol/g protein.

Total SOD activity was measured with a hydroxylamine-based kit (Elabscience, Cat. No. E-BC-K019-M, Houston, TX, USA) at a 1:150 dilution. Absorbance was recorded at 550 nm, and results are expressed as U/mg protein.

TAS and TOS levels were determined colorimetrically using specific kits (Rel Assay Diagnostics, Gaziantep, Turkey). TAS results are expressed as mmol Trolox equivalent/L, and TOS as μmol H_2_O_2_ equivalent/L.

### 4.8. Histopathological Evaluation

Kidney tissues were fixed in 10% neutral buffered formaldehyde for a minimum of 24 h. Following standard dehydration and paraffin embedding, 4 µm-thick sections were cut using an automated microtome. Sections were stained with hematoxylin and eosin (H&E) and evaluated under a light microscope.

The histopathological examination assessed six morphological parameters: tubular epithelial loss, tubular dilatation, intraluminal cast formation, glomerular degeneration, interstitial inflammation, and vacuolization. Each parameter was scored on a semi-quantitative scale from 0 to 3 (0: absent, 1: mild, 2: moderate, 3: severe) [45]. The evaluation was performed by a pathologist blinded to the experimental groups.

### 4.9. Ethical Approval

Approval for the study was received from the Balıkesir University Animal Experiments Ethics Committee (Decision No: 2025/7-2).

### 4.10. Statistical Evaluation

Statistical analyses were performed using SPSS version 23 (IBM Corp., Armonk, NY, USA). Data are expressed as mean ± standard deviation. The normality of data distribution was assessed using normality tests. Normally distributed numerical data were evaluated using one-way ANOVA for multiple group comparisons. When significant differences were detected, post hoc tests (Tukey or Tamhane’s T2, depending on Levene’s test for equality of variances) were performed. For non-normally distributed data, the Kruskal–Wallis test was used for multiple comparisons, followed by the Mann–Whitney U test for pairwise comparisons. A *p*-value < 0.05 was considered statistically significant.

## 5. Conclusions

In conclusion, our results demonstrate that NAS significantly ameliorates the histopathological manifestations of cisplatin-induced renal damage. While systemic biochemical changes were limited, the targeted protection of tubular and glomerular architecture is highly promising. Considering the unique TrkB-mediated pro-survival signaling and direct antioxidant properties of NAS, these findings warrant further investigation into its precise mechanisms as a therapeutic agent for acute kidney injury.

## Figures and Tables

**Table 1:** MDA (μmol/gprotein), SOD (U/mgprotein), TOS (μmol H_2_O_2_ Equiv./L), TAS (mmol Trolox Equiv./L), Urea (mg/dL), and Creatinine (mg/dL) values in the study groups.

	Group C	Group S	Group NAS	Group CP	Group CP + NAS
MDA	0.41 ± 0.06	0.47 ± 0.05	0.52 ± 0.12	0.58 ± 0.05	0.57 ± 0.14
SOD	181.46 ± 66.25	139.91 ± 37.12	155.22 ± 33.64	103.04 ± 15.92	121.19 ± 26.73
TOS	0.11 ± 0.02	0.11 ± 0.01	0.12 ± 0.02	0.13 ± 0.03	0.12 ± 0.02
TAS	0.05 ± 0.01	0.04 ± 0.01	0.04 ± 0.004	0.035 ± 0.004	0.037 ± 0.007
Urea	22.49 ± 10.63	34.58 ± 5.27	37.16 ± 10.73	79.75 ± 44.84	58.17 ± 25.07
Creatinine	0.69 ± 0.03	0.75 ± 0.03	0.71 ± 0.04	0.90 ± 0.10	0.89 ± 0.07

**Table 2:** Total values of the scores obtained in pathological examinations in each group. Tubular epithelial loss, tubular dilatation, intraluminal cast formation, glomerular degeneration, interstitial inflammation, and vacuolization were evaluated. Each parameter was scored on a semi-quantitative scale from 0 to 3 (0: absent, 1+: mild, 2+: moderate, 3+: severe).

	Group C	Group S	Group NAS	Group CP	Group CP + NAS
Tubular epithelial loss	0	0	1	14	6
Tubular dilatation	0	0	0	13	8
Intraluminal cast formation	0	0	1	10	7
Glomerular degeneration	0	0	0	17	6
Interstitial inflammation	0	1	1	15	7
Vacuolization	0	0	0	13	6

## Data Availability

The data presented in this study are available on request from the corresponding author.
